# Noninvasive Tracking of Encapsulated Insulin Producing Cells Labelled with Magnetic Microspheres by Magnetic Resonance Imaging

**DOI:** 10.1155/2016/6165893

**Published:** 2016-08-18

**Authors:** Vijayaganapathy Vaithilingam, Mandy M. W. Yim, Jayne L. Foster, Timothy Stait-Gardner, Jose Oberholzer, Bernard E. Tuch

**Affiliations:** ^1^Commonwealth Scientific and Industrial Research Organization, Future Manufacturing Flagship, North Ryde, NSW 2113, Australia; ^2^Diabetes Transplant Unit, Prince of Wales Hospital, Randwick, NSW 2031, Australia; ^3^School of Medical Sciences, Faculty of Medicine, University of New South Wales, Randwick, NSW 2031, Australia; ^4^Nanoscale Organisation and Dynamics Group, School of Science and Health, University of Western Sydney, Campbelltown, NSW 2560, Australia; ^5^Department of Surgery, University of Illinois at Chicago, Chicago, IL 60612, USA; ^6^School of Biomedical Science, Discipline Physiology, University of Sydney, Sydney, NSW 2006, Australia

## Abstract

Microencapsulated islets are usually injected free-floating into the peritoneal cavity, so the position of the grafts remains elusive after transplantation. This study aims to assess magnetic resonance imaging (MRI) as a noninvasive means to track microencapsulated insulin producing cells following transplantation. Encapsulated insulin producing cells (MIN6 and human islets) were labelled with magnetic microspheres (MM), assessed for viability and insulin secretion, and imaged* in vitro *using a clinical grade 3 T MRI and* in vivo* using both clinical grade 3 T and research grade 11.7 T MRI. Fluorescent imaging demonstrated the uptake of MM by both MIN6 and human islets with no changes in cell morphology and viability. MM labelling did not affect the glucose responsiveness of encapsulated MIN6 and islets* in vitro*.* In vivo *encapsulated MM-labelled MIN6 normalized sugar levels when transplanted into diabetic mice.* In vitro *MRI demonstrated that single microcapsules as well as clusters of encapsulated MM-labelled cells could be visualised clearly in agarose gel phantoms.* In vivo* encapsulated MM-labelled MIN6 could be visualised more clearly within the peritoneal cavity as discrete hypointensities using the high power 11.7 T but not the clinical grade 3 T MRI. This study demonstrates a method to noninvasively track encapsulated insulin producing cells by MM labelling and MRI.

## 1. Introduction

Microencapsulating pancreatic islets are a strategy being investigated to overcome the immune response without the need for toxic immunosuppressive drugs. Traditionally, the islets are encapsulated within alginate hydrogels and have been successfully shown to normalize blood glucose levels in various diabetic preclinical models [1]. However, such success has yet to be achieved in a clinical setting. Phase 1 clinical trials by our group and others have demonstrated that allografting microencapsulated human islets was safe but provided only a minor and transient clinical benefit [2, 3]. Laparoscopic reexamination of a recipient at 16 months after transplantation revealed microcapsules attached to organs and parietal peritoneum, with intact microcapsules surrounded by fibrous tissue containing necrotic islets [3]. Similar results were seen by a Belgium group 3 months after transplantation even in the presence of immunosuppression [4]. Reasons for graft failure are many and may be attributed to either hypoxia or inflammation and erroneous delivery of microcapsules resulting in capsule aggregation leading to islet starvation and death [2, 5–7]. Strategies could be developed to improve clinical outcomes if microencapsulated islets infused into the peritoneal cavity could be tracked by noninvasive means to better understand the optimal delivery method, capsule distribution, and engraftment.

Magnetic resonance imaging (MRI) is the most commonly used noninvasive technique for tracking cells due to its high resolution and enhanced tissue contrast [8]. A range of iron oxide nanoparticles have been employed as MRI contrast agents and especially superparamagnetic iron oxide (SPIO) particles have been extensively studied due to their high relaxivity and enhanced negative contrast [9]. Previous studies have shown that labelling islets with SPIO did not affect viability and labelled islets can be visualised* in vivo *after transplantation [10–13]. Furthermore, a SPIO agent such as Feridex® has been used to produce magnetocapsules for encapsulation of pancreatic islets and for noninvasive tracking by MRI [14]. To date, all studies that have used iron contrast agents have utilised the nanometer-sized SPIO particles. However, there are major drawbacks with SPIO particles in terms of stability and magnetic sensitivity thereby requiring a large number of particles for efficient detection [15]. These drawbacks of SPIO particles can be overcome by using large micrometer-sized iron particles such as magnetic microspheres (MM).

MM are typically larger in size (~1 *μ*m) compared to SPIO particles (~60 to 180 nm) with high iron content per particle, thereby creating a greater magnetic moment and hence enabling efficient detection by MRI [16]. Furthermore, it has been demonstrated that MM exhibit increased relaxation compared to SPIO despite having the equivalent iron content and a single MM can be detected by MRI at a resolution of 100 *μ*m [17–20]. Thus, high iron content, increased sensitivity, and reduced partial volume effects of MM will allow the possibility of detecting cells containing very few MM particles or smaller numbers of MM-labelled cells. Varied cell types such as glioma cells [21], hepatocytes [22], and macrophages [23] are reported to internalise MM and be labelled efficiently without compromising cellular integrity, viability, or function. However, to our knowledge, there are no such reports with insulin producing cells. So, in this study, we explored the feasibility of labelling insulin producing cells (MIN6 and human islets) with MM and investigated the effects on cell viability and function. As a proof of principle study, we also explored the possibility to noninvasively track MM-labelled cells encapsulated within alginate hydrogels both* in vitro* and* in vivo* by MRI.

## 2. Materials and Methods

### 2.1. Tissue Culture

*Human Islets*. Human islets were isolated at the Cell Isolation Laboratory of the University of Illinois at Chicago, USA, and shipped to Sydney as described previously [24]. The islets were then cultured for a day in supplemented CMRL-1066 medium (Mediatech Herndon, VA) containing 1.5% human albumin at 37°C in 5% CO_2 _before being encapsulated. All procedures relating to isolating human islets and obtaining them were approved by the Human Research Ethics Committee of the University of Illinois at Chicago and the University of New South Wales, respectively.

*MIN6*. The mouse insulinoma beta cell line (MIN6) was cultured in Dulbecco's Modified Eagle's Medium (DMEM) (Gibco, Carlsbad, CA) containing 10% fetal bovine serum (FBS) (Gibco) and 1% penicillin and streptomycin solution (Gibco) at 37°C in 5% CO_2_.

### 2.2. Magnetic Microsphere (MM) Labelling

The MM were 0.9 *μ*m superparamagnetic styrene-divinylbenzene inert polymer microspheres that contained a magnetite core and a fluorescein-5-isothiocyanate dye (Dragon Green) encapsulated within the cross-linked polymer sphere (Bangs Laboratories, Fishers, IN). MIN6 and human islets were labelled by culturing them in culture media supplemented with 1% v/v gamma irradiated MM for 24 hr with each mL of MM containing 6.2 mg iron oxide. To test for MM uptake, MM-labelled cells were viewed under a fluorescent microscope (ZeissAxioskop 2, Berlin, Germany) and iron content was determined semiquantitatively by counting the number of magnetic microspheres within labelled cells stained with haematoxylin and eosin and using the analysis data supplied by the company (Table 1).

### 2.3. Viability

Viability of unlabelled and MM-labelled MIN6 and human islets was determined using fluorescent dyes 6-carboxyfluorescein diacetate (6-CFDA, Sigma, St. Louis, MO) and propidium iodide (PI, Sigma) as described previously [25]. The cells and islets were visualised using a 450–490 nm filter for 6-CFDA and a 510–560 nm filter for PI. The number of green (live) cells and red (dead) cells was separately assessed and the percentage of viable cells was then determined.

### 2.4. Insulin Secretion

*MIN6. *A static incubation assay was carried out to assess insulin secretion of unlabelled and MM-labelled MIN6 cells, as described previously [26]. Briefly, the cells were seeded onto 6-well plates and incubated in the basal media of HEPES buffered Earle's Medium containing 0.2% bovine serum albumin (2.8 mM glucose) initially to stabilise insulin secretion followed by exposure to either basal (2.8 mM glucose) or stimulation media (20 mM glucose) for 1 hr and supernatants collected for insulin measurement by radioimmunoassay (RIA) (rat insulin RIA kit, LINCO Research, MO).

*Human Islets*. Aliquots of unlabelled and MM-labelled human islets were exposed to either 2.8 mM (basal) or 20 mM (stimulus) of glucose for 1 hr at 37°C and supernatant collected for human insulin measurement by radioimmunoassay (RIA) (human insulin RIA kit, LINCO Research) as described previously [24].

### 2.5. Encapsulation

The unlabelled and MM-labelled MIN6 and human islets were encapsulated as described previously [25]. The average size of the microcapsules was 496.5 ± 37.1 *μ*m (range 431–567 *μ*m, median 494 *μ*m). Empty microcapsules containing MM were synthesised as above by mixing 30 *μ*L of MM with 0.4 mL of 2.2% alginate solution (UPMVG Pronova, FMC Biopolymer, Sandvika, Norway). The number of cells within the capsule was determined by calculating the total number of cells divided by the total number of capsules produced per run as reported previously [27]. Accordingly, it was estimated that each microcapsule contained approximately 6 × 10^4^ cells. Cell viability and insulin secretion of encapsulated insulin producing cells were determined as described above. For MIN6, static stimulation was carried out on microcapsules containing 1 × 10^6^ cell equivalents per sample and performed in triplicate. For human islets, 20 encapsulated islets were individually hand-picked each for basal and stimulus conditions and performed in triplicate.

### 2.6. Transplantation

All animal experimental procedures were approved by the “Animal Care and Ethics Committee” of the University of New South Wales and Commonwealth Scientific and Industrial Research Organization (CSIRO), North Ryde, Australia. Transplantation of encapsulated unlabelled and MM-labelled MIN6 cells was carried out as described previously [24]. Briefly, nonfasting male C57BL/6 mice (6–8 weeks) were made diabetic (three consecutive blood glucose levels (BGL) > 15 mmol/L) by streptozotocin (275 mg/kg body weight) (Alexis Biochemicals, Lausen, Switzerland) and infused into the peritoneal cavity with (i) encapsulated unlabelled MIN6 cells (1.5 × 10^6^ cells/mouse; *n* = 4), (ii) encapsulated MM-labelled MIN6 cells (1.5 × 10^6^ cells/mouse; *n* = 4), and (iii) empty capsules (*n* = 4). The BGL and weights were measured and animals were considered normoglycemic if three consecutive BGL of < 10 mmol/L were recorded and an oral glucose tolerance test (OGTT) was carried out. At the end point, the capsules were retrieved by peritoneal lavage and BGL were monitored for a further few days. Capsules were observed under the microscope for signs of overgrowth and/or breakage.

### 2.7. Magnetic Resonance Imaging (MRI)

#### 2.7.1. *In Vitro*

*In vitro* MRI of encapsulated MM-labelled cells was performed using a Philips Achieva 3 T clinical grade MRI machine (Philips Medical Systems, Eindhoven, Netherlands). The samples were fixed in 10% buffered formalin (Sigma) and embedded in 2% agarose (Sigma) in eppendorf tubes. These tubes were placed inside a 14 × 46 × 25 cm SENSE-4 wrist coil (Invivo, WI) for excitation and detection. Two different imaging sequences were used to create two different types of contrast images: T1-weighted images and T2^*∗*^-weighted images. 3D T2^*∗*^ fast field echo (FFE) gradient sequences were applied to acquire T2^*∗*^-weighted images using the following parameters: slice thickness of 0.5 mm, time of repetition (TR) = 30 ms, echo time (TE) = 13.81 ms, number of slices = 50, field of view (FOV) = 70 mm, and 256 × 128 imaging matrix. T1-weighted images were obtained using a T1 turbo spin echo (TBE) sequence with the following image parameters: slice thickness of 1 mm, slice separation of 0.1 mm, TR = 420 ms, TE = 12 ms, number of slices = 25, FOV = 70 mm, and 256 × 128 imaging matrix. Imaging planes were transversal. The following samples were scanned using the clinical grade 3 T MRI: (i) 50 *μ*L MM-labelled MIN6 capsules; (ii) 50 *μ*L unlabelled MIN6 capsules; (iii) 50 *μ*L encapsulated MM; (iv) a single capsule of MM-labelled MIN6; (v) single capsule of encapsulated MM; (vi) 50 *μ*L encapsulated MM-labelled human islets; (vii) a single capsule of encapsulated MM-labelled human islet. A 1 × 1 mm area of the 2% agarose also was embedded with 3 *μ*L and 1 *μ*L of MM, respectively, as positive controls.

#### 2.7.2. *In Vivo*

Initial* in vivo *scans were performed using the Philips Achieva 3 T clinical grade MRI machine (Philips Medical System) and a specialised magnetic resonance coil. Two different coils were used for detection: the SENSE-4 wrist coil (Invivo) and a 37 × 25 × 17 cm mouse coil (Philips). Both coils used the 3D T2^*∗*^ FFE gradient sequence to form T2^*∗*^-weighted images. The SENSE-4 wrist coil used the imaging parameters outlined in the above section. The mouse coil scanned the mice using the following parameters: slice thickness = 0.5 mm, TR = 30 ms, TE = 14 ms, number of slices = 50, FOV = 50 mm × 60 mm, and 240 × 260 imaging matrix. At day 1 after transplant, the mice were anaesthetised with 70 mg/kg sodium pentobarbitone (Virbac Animal Health), scanned with a gel heat pack when using the SENSE-4 wrist coil, and scanned for T2^*∗*^-weighted images. To minimise the number of artefacts within the image, subsequent scans were taken after the mice were given 50% v/v glucose solution 15 or 40 min before the scan by oral gavage with the antispasmodic agent Buscopan® (Boehringer Ingelheim, Ingelheim, Germany) administered intraperitoneally before the MRI scan.

To enhance resolution and get better quality images, subsequent scans were carried out using the vertical Avance II wide-bore 11.7 T MRI scanner (Bruker, Germany) at the Biomedical Magnetic Resonance Facility (University of Western Sydney, Campbelltown, Australia). The mice experiments were conducted with the Mini-0.75 animal probe which is capable of generating gradients of 0.45 Tm^−1^. The abdominal cavities of both mice transplanted with encapsulated unlabelled (*n* = 3; 1.5 × 10^6^ cells/mouse) and MM-labelled MIN6 (*n* = 3; 1.5 × 10^6^ cells/mouse) were imaged on the day of transplant with a standard gradient echo (FLASH) sequence with 1 mm slice thickness, matrix size of 256 × 256, and ~200 *μ*m in-plane isotropic voxels; the repetition time was 100 ms and echo time was 6 ms, although microcapsule clusters were visible under a range of repetition and echo times. All mice were scanned with the same parameters.

### 2.8. Statistical Analysis

All data were expressed as mean ± SEM. One-way analysis of variance was used to compare data among groups and Student's* t*-test was used to compare data between the groups. The results were considered significant when *p* < 0.05. All statistical analysis was performed using the GraphPadInStat software (GraphPad Software, La Jolla, CA).

## 3. Results

### 3.1. Magnetic Microsphere (MM) Labelling and Encapsulation

**Figure 1:**
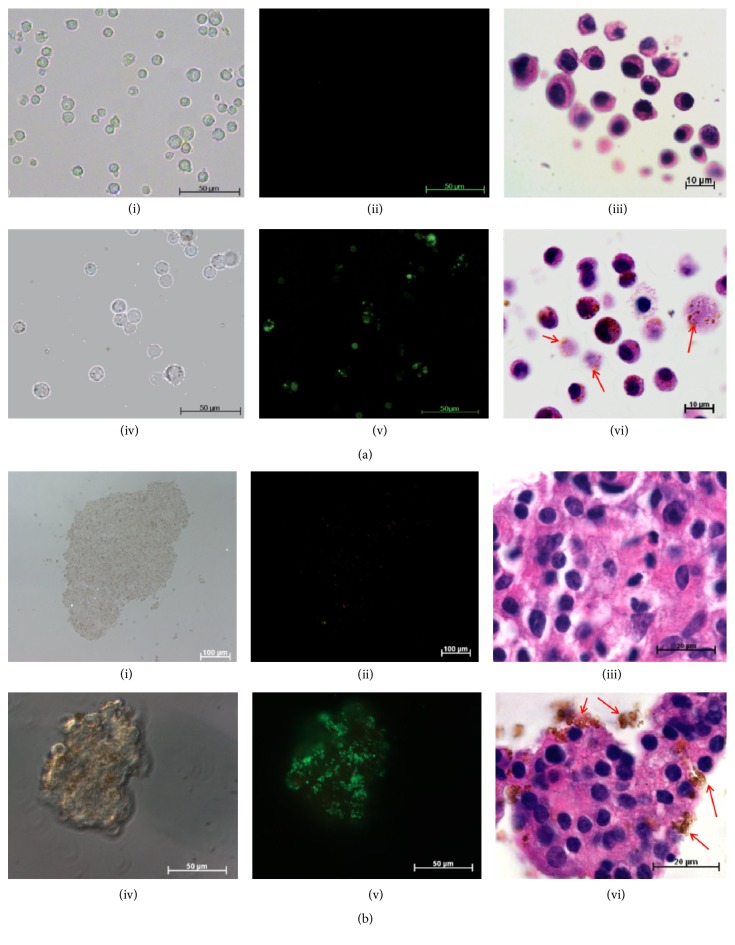
MM labelling. MM are readily taken up by both MIN6 (a) and human islets (b) by simple incubation for 24 hr in culture as evident from the phase contrast (a and b; iv), fluorescent (a and b; v), and histology (a and b; vi) images compared to unlabelled cells (a and b; i, ii, and iii). MM are seen as brown spots (arrows) and are scattered throughout the cytoplasm of both MIN6 (a; vi) and human islets (b; vi). Bar is 50 *μ*m for (a) (i, ii, iv, and v) and (b) (iv and v), 100 *μ*m for (b) (i and ii), 10 *μ*m for (a) (iii and vi), and 20 *μ*m for (b) (iii and vi).

**Figure 2:**
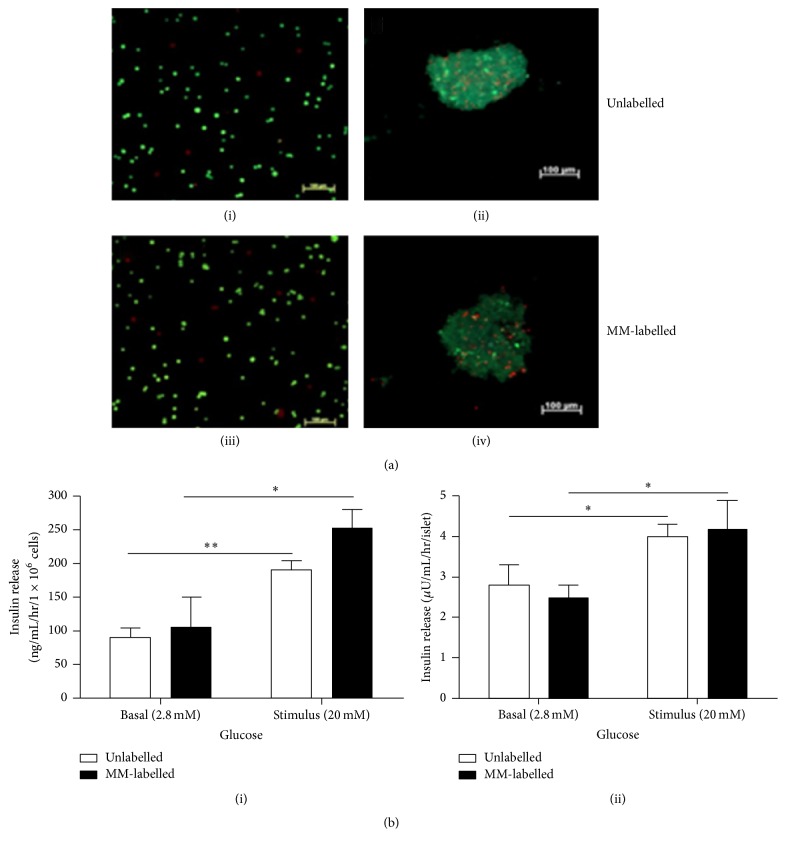
Viability and insulin secretion of nonencapsulated MM-labelled cells. (a) Viability images of unlabelled and MM-labelled MIN6 (i and iii) and human islets (ii and iv), respectively. Bar is 50 *μ*m for (i) and (iii) and 100 *μ*m for (ii) and (iv). Viability is estimated as a percentage of green (live cells) to red (dead cells) fluorescence. Values are mean ± SEM (*n* = 3 aliquots for MIN6 and *n* = 100 clusters for islets). (b) Glucose stimulated insulin secretion of both MIN6 (i) and human islets (ii) 24 hr after MM labelling. Values are mean ± SEM (*n* = 3); ^*∗∗*^ *p* < 0.01 and ^*∗*^ *p* < 0.05 for basal versus stimulated insulin secretion for both unlabelled and MM-labelled MIN6 and human islets, respectively (Student's* t*-test).

**Figure 3:**
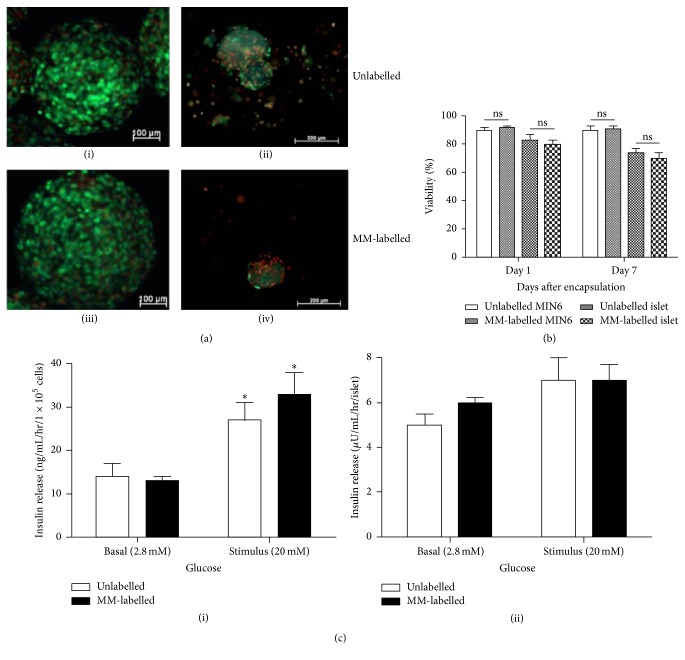
Viability and insulin secretion of encapsulated MM-labelled cells. (a) Viability images of unlabelled and MM-labelled encapsulated MIN6 (i and iii) and human islets (ii and iv), respectively. Bar is 100 *μ*m for (i) and (iii) and 200 *μ*m for (ii) and (iv), respectively. (b) Viability of encapsulated MM-labelled cells cultured for varied time points after encapsulation. Values are mean ± SEM (*n* = 100 capsules for each time point); ns, *p* > 0.05 for viabilities between unlabelled and MM-labelled MIN6 and human islets at each time point (Student's* t*-test). (c) Glucose stimulated insulin secretion of both MM-labelled MIN6 (i) and human islets (ii) 24 hr after encapsulation. Values are mean ± SEM (*n* = 3); ^*∗*^ *p* < 0.05 for basal versus stimulated insulin secretion for encapsulated unlabelled and MM-labelled MIN6, respectively (Student's* t*-test).

Incubating MIN6 cells and human islets with MM for 24 hr suggests that the cells readily take up the iron oxide microspheres and are effectively labelled as detected by fluorescent microscopy. No change in cell morphology was observed between unlabelled and MM-labelled cells and the MM were seen scattered throughout the cell cytoplasm (Figures 1(a) and 1(b)). The viability of MIN6 and human islets was not affected at >95% and 83 ± 1%, respectively, 24 hr after labelling, similar to unlabelled cells (>95% and 85 ± 1% for MIN6 and human islets, respectively; *p* > 0.05) (Figure 2(a)). There were no differences in cell viabilities between MIN6 cells cultured at varied concentrations (0.25%, 0.5%, and 1% v/v MM) suggesting the nontoxic nature of MM (data not shown). Furthermore, MM labelling did not affect cell function and MM-labelled MIN6 and human islets responded to high glucose with a stimulation index of 2.4 and 1.7, respectively, similar to unlabelled cells (Figure 2(b)). Encapsulation of MM-labelled MIN6 and human islets within barium alginate microcapsules affected neither viability nor function. The viability of MM-labelled MIN6 cells was ~90% after encapsulation similar to unlabelled cells and they remained so for at least a week in culture (Figures 3(a) and 3(b)). Similarly, there was no significant difference in the viability of both encapsulated MM-labelled and unlabelled human islets cultured for days 1 and 7, respectively (Figures 3(a) and 3(b)). Furthermore, there was no difference in the viabilities of encapsulated MM-labelled and unlabelled MIN6 cells cultured for 14 (92.4 ± 1% versus 91.8 ± 1.1%) and 21 (87 ± 1.3% versus 88.8 ± 1.1%) days, respectively, with only a slight reduction in viability seen in both groups at day 21. There was no significant difference in the glucose response of encapsulated MM-labelled MIN6 and human islets with a stimulation index of 2.5 and 1.2, respectively, similar to encapsulated unlabelled cells (1.9 and 1.4 for unlabelled MIN6 and human islets, resp.) (Figure 3(c)). The iron content within each microcapsule was calculated based on the number of MM taken up by the cells and iron content within each microsphere. Accordingly, it was found that each MIN6 cell had internalised ~8 pg of iron and each microcapsule had an iron content of approximately ~50 ng (Table 1). To determine whether MM can leak out through the pores of microcapsules, MM were encapsulated in alginate microcapsules and incubated in culture media for various time periods. The MM remained within the microcapsules and no MM were found in the surrounding media for at least 60 days in culture suggesting that MM are trapped within the polymer framework of alginate capsules (Supplementary Figure 1 in Supplementary Material available online at http://dx.doi.org/10.1155/2016/6165893).

### 3.2. Transplantation of Encapsulated MM-Labelled Cells

**Figure 4:**
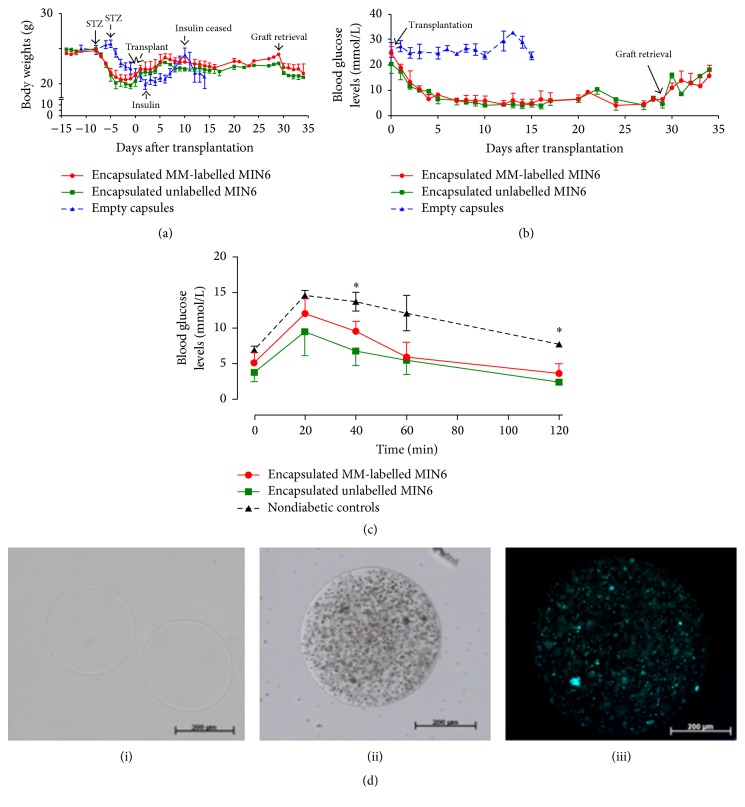
Transplantation of encapsulated MM-labelled MIN6 cells. Diabetic mice were transplanted with either encapsulated MM-labelled or unlabelled MIN6 (1.5 × 10^6^ cells/mouse) into the peritoneal cavity and monitored for up to 30 days after transplantation. (a) Body weights (g) of animals transplanted with either encapsulated MM-labelled or unlabelled MIN6 cells and empty microcapsules. Values are mean ± SEM (*n* = 4 in each group). Broken arrows denote the group transplanted with empty microcapsules. (b) Blood glucose levels (BGL) of mice transplanted with either encapsulated MM-labelled or unlabelled MIN6 cells and empty microcapsules. Normoglycemia was achieved in 100% of mice receiving both encapsulated MM-labelled and unlabelled MIN6 cells. Values are mean ± SEM (*n* = 4 in each group). (c) OGTT done at day 16 after transplantation after normalization of BGL. Values are mean ± SEM (*n* = 3 for encapsulated MM-labelled and unlabelled MIN6  and  *n* = 4 for nondiabetic controls); ^*∗*^ *p* < 0.05 at 40 and 120 min, respectively, at 40 min nondiabetic control > encapsulated unlabelled MIN6 and at 120 min nondiabetic control > encapsulated MM-labelled and unlabelled MIN6 (one-way ANOVA with* post hoc *Tukey-Kramer Multiple Comparison test). (d) Graft retrieval from mice transplanted with empty microcapsules at day 15 (i) and encapsulated MM-labelled MIN6 at day 29 (ii). Fluorescently labelled MM can still be detected on retrieved grafts at day 29 after transplantation (iii). Bar is 200 *μ*m for (i), (ii), and (iii), respectively.

After STZ induction the body weights of the animals dropped significantly with a concomitant rise in BGL and became diabetic with a mean BGL of 23.5 ± 1.9 mmol/L (Figures 4(a) and 4(b)). All mice transplanted with encapsulated MM-labelled and unlabelled MIN6 became normoglycemic by 5.4 ± 0.6 days after transplantation (median: 5 days; range: 3–8 days) (Figure 4(a)). BGL declined from 21.8 ± 2.6 to 7.3 ± 0.7 mmol/L and the BGL remained constant until day 29 (5.6 ± 0.8 mmol/L) (Figure 4(b)) with the animals regaining their lost body weights. OGTT carried out at day 16 after transplantation suggested that animals transplanted with encapsulated MM-labelled and unlabelled MIN6 handled glucose normally similar to or better than nondiabetic controls (Figure 4(c)). To determine whether the lowering of BGL was due to the transplanted encapsulated cells and not from residual pancreatic beta cells, the grafts were retrieved at day 29 by a peritoneal lavage, with an 89% success rate. Immediately after graft retrieval, the BGL started to rise and continued so till day 34 when the BGL was 16.7 ± 2.4 mmol/L which was accompanied by a concomitant drop in body weights to 21.1 ± 0.6 g (Figures 4(a) and 4(b)). However, diabetic animals transplanted with empty microcapsules remained hyperglycaemic throughout and required daily insulin injections (0.5 units of Glargine, subcutaneously) to maintain their body weights until day 15 when they were euthanized (Figure 4(b)). Microscopic examination of retrieved grafts showed that microcapsules were intact and free of fibrotic overgrowth and importantly MM could still be detected within microcapsules of labelled MIN6 cells (Figure 4(d)).

### 3.3. *In Vitro* MRI

**Figure 5:**
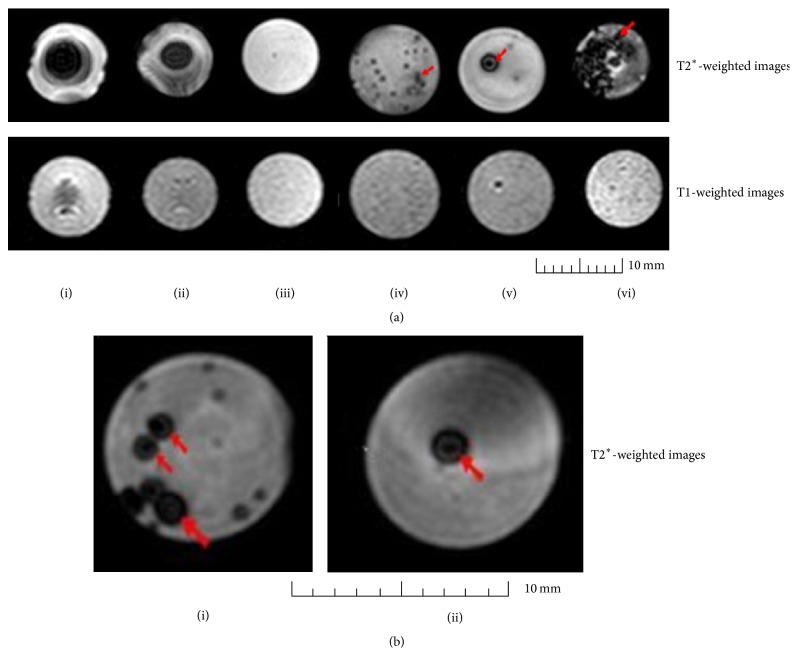
*In vitro* MRI of encapsulated MM-labelled cells by 3 T scanner. (a) T2^*∗*^- and T1-weighted images of 3 *μ*L (i) and 1 *μ*L (ii) of naked MM solution; encapsulated unlabelled MIN6 (iii); encapsulated MM alone (iv); single capsule of encapsulated MM-labelled MIN6 (v); and clusters of encapsulated MM-labelled MIN6 (vi). (b) T2^*∗*^-weighted images of clusters (i) and single capsule (ii) of encapsulated MM-labelled human islets. The red arrows point to the discrete hypointense spots created on the agarose phantoms by MM.

In order to determine if MM could be detected by MRI, a variety of samples were embedded in 2% agarose gel phantoms and scanned using two different scanning modes: T1-weighted images and T2^*∗*^-weighted images using a clinical grade 3 T MRI scanner. Each type of scanning used different sequences and parameters to visualise the samples with different contrasts. Initially to determine whether the naked MM could be detected by MRI, 1 *μ*L and 3 *μ*L of MM solution, containing 6.2 and 18.6 *μ*g iron oxide, respectively, encompassed within agarose gel phantoms were imaged. As expected, MRI scans of the sample containing 3 *μ*L MM had a greater signal loss than that of 1 *μ*L MM, and the resulting T2^*∗*^-weighted image of 3 *μ*L MM showed a strong hypointensity with a more pronounced darker spot than 1 *μ*L MM (Figures 5(a)(i) and 5(a)(ii)). Encapsulated unlabelled MIN6 could not be detected in T2^*∗*^-weighted images in contrast to encapsulated MM alone being detectable as discrete hypointense dark spots (Figures 5(a)(iii) and 5(a)(iv)). Encapsulated MM-labelled MIN6 cells had a greater signal loss than encapsulated MM alone, appearing as strong dark hypointense spots (Figure 5(a)(vi)). The 3 T scanner was even able to detect a single microcapsule of encapsulated MM-labelled MIN6 cells, and T2^*∗*^-weighted image appeared as a single defined hypointense dark spot (Figure 5(a)(v)). However, using T1-weighted images of the same samples we were unable to visualise clearly either naked or encapsulated MM as well as the encapsulated MM-labelled cells. Only the samples that had the strongest signal loss in T2^*∗*^-weighted images could be detected but with very poor resolution. Similarly, single as well as clusters of encapsulated MM-labelled human islets also could be visualised clearly as discrete hypointense spots in T2^*∗*^-weighted images using the clinical grade 3 T MRI scanner (Figure 5(b)).

### 3.4. *In Vivo* MRI

For* in vivo* MRI, initial scans were performed on the clinical grade 3 T scanner on mice transplanted with encapsulated MM-labelled MIN6. Microcapsules containing MM-labelled MIN6 could not be visualised in the T2^*∗*^-weighted images as the peritoneal cavity was filled with artefacts, particularly air, which also appeared as hypointense regions in the image (Supplementary Figure 2A). To overcome the artefacts, minimise air within the bowel, and eliminate gastrointestinal movement, we administered 50% glucose solution as a positive contrast for bowel distension and Buscopan as an antispasmodic agent to reduce peristaltic movement. The quality of scans improved significantly, with the greatest reduction of air within the bowel occurring when 50% glucose was administered 40 min before the scan by oral gavage and Buscopan injected intraperitoneally immediately before the scan. This procedure greatly reduced the artefacts and encapsulated MM-labelled MIN6 appeared as dark hypointense spots which were seen at the surface of abdomen in the T2^*∗*^-weighted images (Supplementary Figure 2B). However, the resolution was not clear and the image had a low signal-to-noise ratio.

**Figure 6:**
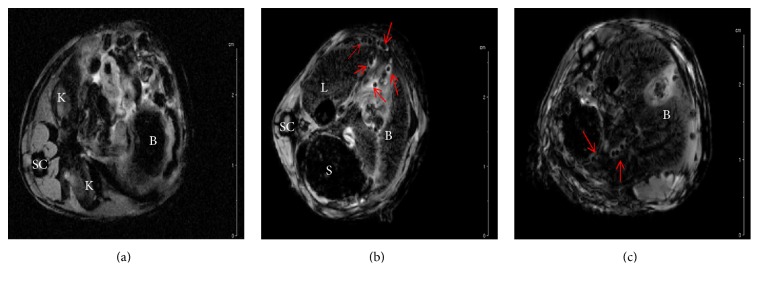
*In vivo* MRI of encapsulated MM-labelled cells by 11.7 T scanner. MR images of the abdominal cavity of mice on the day they were transplanted with encapsulated unlabelled MIN6 (a) and encapsulated MM-labelled MIN6 (b and c). The arrows point to single as well as clusters of encapsulated MM-labelled MIN6 which appear as discrete hypointensities and are spread throughout the peritoneal cavity. The letters on the figure identify different organs as follows: B is for bowel, K is for kidney, L is for liver, S is for spleen, and SC is for spinal cord.

To enhance resolution and increase signal-to-noise ratio we used a high power 11.7 T MRI scanner. With this more sophisticated scanner, encapsulated MM-labelled MIN6 could be visualised clearly within the peritoneal cavity as discrete hypointensities with enhanced resolution and were found scattered throughout the abdominal cavity (Figures 6(a), 6(b), and 6(c) and Supplementary movie files). The greater signal-to-noise ratio and high resolution provided by 11.7 T, but not the clinical grade 3 T MRI scanner, enabled us to noninvasively track single as well as clusters of microcapsules within the peritoneal cavity following transplantation.

## 4. Discussion

To our knowledge, our study is the first study to report the use of MM to label insulin producing cells and noninvasively track encapsulated MM-labelled cells* in vivo* by MRI. MM have been used previously for the detection of varied cell types such as hepatocytes [22], macrophages [23], and stem cells [16]. All these studies demonstrated that labelling with MM did not alter cell morphology, viability, or their differentiation potential. In our study we found that insulin producing cells MIN6 and human islets can be iron labelled efficiently by simple overnight coincubation with MM with an estimated iron uptake of ~8 pg iron per cell. This is in agreement with previously published reports using SPIO particles where the nonspecific iron uptake varied between 2 and 12 pg/cell with islets [28] and between 10 and 20 pg/cell for other cell types [29]. Moreover, in a study utilising SPIO particles, even with cationic transfection agent poly-l-lysine coupled with electroporation resulted in an iron content of only 1.72 pg/cell [13] suggesting that MM are taken up efficiently and nontoxically by insulin producing cells without the aid of transfection agents. There was no obvious difference in cell morphologies between MM-labelled and unlabelled cells and MM were seen scattered throughout the cytoplasm. In the human islets the MM were more concentrated within the cytoplasm at the periphery rather than at the centre of the islets, as has been reported by others using SPIO particles [13]. MM used in our study were ~1 *μ*m and have a hydrophobic styrene/divinyl benzene outer shell which might have allowed for substantial cell membrane interactions resulting in enhanced endocytosis and hence efficient labelling. Furthermore, the inert polystyrene coating of MM makes it nonbiodegradable compared to dextran-coated SPIO particles [11] thereby allowing MM to be tracked* in vivo *for long periods of time, with >100 days being reported in one study [20]. Labelling with MM did not alter the viability of human islets or MIN6 suggesting the nontoxic nature of MM similar to SPIO. There are conflicting results in the literature on the effects of SPIO labelling on glucose stimulated insulin secretion* in vitro* with studies showing no differences in insulin secretion between unlabelled and labelled cells [12, 28, 30] but one study reported a 50% reduction in insulin secretion in labelled cells [11]. In our study, MM labelling did not affect insulin secretion of either MIN6 or human islets further confirming the nontoxic nature of micrometer-sized MM. However, the low stimulation index of both the labelled and unlabelled human islets suggests that their functioning capacity was not ideal.

Next we explored the possibility of encapsulating MM-labelled cells within alginate hydrogels and tested their viability and function both* in vitro* and* in vivo*. The labelled cells were encapsulated because this technique of immunoisolation is thought to be a means of implanting cells into recipients for therapeutic purposes, without the need to administer immunosuppressive drugs. We found that MM-labelled human islets and MIN6 could be encapsulated within barium alginate capsules and remained viable for at least 1 week in culture, without diminution of ability to secrete insulin* in vitro*. Furthermore, encapsulated MM-labelled MIN6 were able to normalize blood glucose levels when transplanted into diabetic immunocompetent recipients similar to unlabelled cells. These results were similar to the outcome of another study where *β*TC-6 cells encapsulated within magnetocapsules normalized blood glucose levels when transplanted into immunocompetent diabetic mice [14]. However, in that study rather than direct cell labelling, SPIO nanoparticles were complexed within alginate-poly-l-lysine (PLL) microcapsules to produce MRI trackable magnetocapsules with an iron content of 80.8 ± 4.9 ng per capsule. In our study, we managed to get an iron concentration of 50 ± 6 ng per capsule by direct cell labelling without the incorporation of PLL which is immunogenic [31, 32].

Cells labelled with iron nanoparticles appear as dark hypointensities in an MRI image, with a greater content of iron nanoparticles creating a greater “blooming effect” [17, 33]. Our results confirm this with 3 *μ*L MM giving a much larger signal loss than 1 *μ*L MM. In our study we have demonstrated that a 3 T MRI clinical machine was able to clearly detect a single capsule of MM-labelled human islets as well as MIN6 cells* in vitro *using T2^*∗*^-weighted sequences. On the contrary, only a minimum number of 250 nonencapsulated islets could be detected with direct SPIO labelling using a 1.5 T clinical MRI scanner [13]. The higher contrast of MM compared to SPIO might be attributed to the higher iron content of MM confined to a small area which might have facilitated the detection of single microcapsule using a clinical grade 3 T scanner. To monitor transplanted cells over a long period of time, it is essential that the iron particles are retained within the cells to provide sufficient contrast to be detected by MRI. This has been a major problem with iron nanoparticles such as SPIO as it was demonstrated that cell divisions can dilute iron concentrations to values below detectability of 0.1 pg [15, 34]. Furthermore, the iron nanoparticles could sometimes be discharged out of the cells by a process called exocytosis [35] and indirect labelling may occur if labelled cells were phagocytosed by macrophages [12]. In our study, the presence of fluorescently labelled MM within MIN6 cells was monitored over 4 weeks* in vitro *and was found to have decreased from 100% to 30% in nonencapsulated cells after four passages in a 14-day period after initial labelling (data not shown). However, unlike the SPIO particles, dilution of MM over time should not be an issue due to the higher iron content of MM and if cells are encapsulated. Encapsulated MM-labelled MIN6 continued to fluoresce strongly for over 29 days despite proliferation indicating the continual presence of MM within the capsule. Furthermore, we demonstrated that encapsulated MM could remain within microcapsules for at least 60 days* in vitro *without leaching suggesting that MM were trapped within the alginate matrix. The large particle size of MM, ~0.9 *μ*m with molecular weight of ~7.5 × 10^8^ kDa, is considerably larger than the pore size of alginate microcapsules which have a molecular weight cut-off of only ~250 kDa [25]. Furthermore, as the MM and MM-labelled cells are trapped within the microcapsules, they would not be phagocytosed by macrophages outside the microcapsules and accidental macrophage labelling would not occur. These data suggest that encapsulated MM-labelled cells could be monitored by MRI over long periods of time without considerable signal loss compared to SPIO labelling.

MRI of the extensive volume of peritoneal cavity has always been a challenge for radiologists. The small size of the microcapsules compared to the entire area of the peritoneal cavity renders detection of microcapsules extremely difficult when the location of the transplant is unknown. However, labelling with MM facilitated the tracking of individual microcapsules* in vivo*. The MRI scans on the* in vitro *samples showed that a T2^*∗*^-weighted sequence can detect MM, if fast field echo gradient sequence is applied to scan the entire peritoneal cavity. This sequence initiates a fast scan that provides high spatial resolution in extremely thin sections and therefore provides better anatomic coverage [36] than other conventional sequences which have lower resolution but is sufficient for imaging organs. Two other strategies to reduce extraneous noise were required for successful imaging of the encapsulated MM-labelled MIN6 cells. These were the administration of 50% glucose to distend the bowel, thereby reducing the amount of air, which appears black as does MM and the administration of the antispasmodic Buscopan to reduce peristaltic artefacts [36, 37]. To our knowledge, this is the first study to report the use of a clinical grade 3 T MRI for microencapsulated cell tracking* in vivo* using MM. However, the presence of artefacts cannot be completely ignored which made detection of individual microcapsules difficult and sometimes unreliable when imaged using a clinical grade 3 T MRI scanner. To reduce the noise, increase signal, and improve image quality, further scans were carried out using a high resolution small animal 11.7 T MRI scanner. With this instrument, single microcapsules as well as clusters of microcapsules containing MM-labelled cells could be easily tracked as discrete hypointensities spread throughout the peritoneal cavity.

## 5. Conclusions

In conclusion, to our knowledge this is the first proof of principle study to report the use of micron sized iron particles such as MM for labelling insulin producing cells and microencapsulated cell tracking. We have demonstrated that MIN6 and human islets can be labelled efficiently using MM and that encapsulated MM-labelled cells can be imaged noninvasively by MRI. Noninvasive tracking of microencapsulated islets will provide valuable information about capsule distribution and engraftment with time after transplantation, which may subsequently enhance the success rates of microencapsulated islets in a clinical setting. This platform technology of MM labelling, microencapsulation, and cell tracking has huge potential and can be readily applied to other cell based therapies and regenerative medicine.

## Supplementary Material

The entrapment of MM within alginate microcapsules for varied time points, in vivo MRI of encapsulated MM labelled cells by 3T scanner and movie files for in vivo MRI of encapsulated MM labelled cells by 11.7T scanner are provided in the Supplementary Material

## Figures and Tables

**Table 1:** Estimation of iron content of encapsulated MM-labelled cells.

Weight of MM (kDa)^*∗*^	Weight of MM (pg)^*∗*^ (*A*)	Iron oxide (%)^*∗*^ (*B*)	Iron oxide per MM (pg) (*C* = *A* × *B*)	MM per cell (*D*)	Iron content per cell (pg) (*E* = *C* × *D*)	Cells per capsule^†^ (*F*)	Iron content per capsule (ng) (*E* × *F*)
7.5 × 10^8^	1.24	62	0.77	10.95 ± 1	8.43 ± 1	~6000	50 ± 6

MM: magnetic microspheres; values = mean ± SD.

^*∗*^Data obtained from bangs laboratories.

^†^Estimated by calculating the total number of cells divided by the total number of capsules produced.

## References

[1] Silva A. I., Norton De Matos A., Brons I. G., Mateus M. (2006). An overview on the development of a bio-artificial pancreas as a treatment of insulin-dependent diabetes mellitus. *Medicinal Research Reviews*.

[2] Basta G., Montanucci P., Luca G. (2011). Long-term metabolic and immunological follow-up of nonimmunosuppressed patients with type 1 diabetes treated with microencapsulated islet allografts: four cases. *Diabetes Care*.

[3] Tuch B. E., Keogh G. W., Williams L. J. (2009). Safety and viability of microencapsulated human islets transplanted into diabetic humans. *Diabetes Care*.

[4] Jacobs-Tulleneers-Thevissen D., Chintinne M., Ling Z. (2013). Sustained function of alginate-encapsulated human islet cell implants in the peritoneal cavity of mice leading to a pilot study in a type 1 diabetic patient. *Diabetologia*.

[5] De Vos P., Wolters G. H. J., Van Schilfgaarde R. (1994). Possible relationship between fibrotic overgrowth of alginate-polylysine- alginate microencapsulated pancreatic islets and the microcapsule integrity. *Transplantation Proceedings*.

[6] de Vos P., Faas M. M., Strand B., Calafiore R. (2006). Alginate-based microcapsules for immunoisolation of pancreatic islets. *Biomaterials*.

[7] Horcher A., Zekorn T., Siebers U. (1994). Transplantation of microencapsulated islets in rats: evidence for induction of fibrotic overgrowth by islet alloantigens released from microcapsules. *Transplantation Proceedings*.

[8] Grimm J., Kircher M. F., Weissleder R. (2007). Cell tracking. *Radiologe*.

[9] Long C. M., Bulte J. W. M. (2009). *In vivo* tracking of cellular therapeutics using magnetic resonance imaging. *Expert Opinion on Biological Therapy*.

[10] Berkova Z., Kriz J., Girman P. (2005). Vitality of pancreatic islets labeled for magnetic resonance imaging with iron particles. *Transplantation Proceedings*.

[11] Jirák D., Kríz J., Herynek V. (2004). MRI of transplanted pancreatic islets. *Magnetic Resonance in Medicine*.

[12] Kriz J., Jirák D., Girman P. (2005). Magnetic resonance imaging of pancreatic islets in tolerance and rejection. *Transplantation*.

[13] Tai J. H., Foster P., Rosales A. (2006). Imaging islets labeled with magnetic nanoparticles at 1.5 Tesla. *Diabetes*.

[14] Barnett B. P., Arepally A., Karmarkar P. V. (2007). Magnetic resonance-guided, real-time targeted delivery and imaging of magnetocapsules immunoprotecting pancreatic islet cells. *Nature Medicine*.

[15] Shapiro E. M., Skrtic S., Sharer K., Hill J. M., Dunbar C. E., Koretsky A. P. (2004). MRI detection of single particles for cellular imaging. *Proceedings of the National Academy of Sciences of the United States of America*.

[16] Boulland J.-L., Leung D. S. Y., Thuen M. (2012). Evaluation of intracellular labeling with micron-sized particles of iron oxide (MPIOs) as a general tool for in vitro and in vivo tracking of human stem and progenitor cells. *Cell Transplantation*.

[17] Hinds K. A., Hill J. M., Shapiro E. M. (2003). Highly efficient endosomal labeling of progenitor and stem cells with large magnetic particles allows magnetic resonance imaging of single cells. *Blood*.

[18] Shapiro E. M., Skrtic S., Koretsky A. P. (2005). Sizing it up: cellular MRI using micron-sized iron oxide particles. *Magnetic Resonance in Medicine*.

[19] Wu Y. L., Ye Q., Foley L. M. (2006). *In situ* labeling of immune cells with iron oxide particles: an approach to detect organ rejection by cellular MRI. *Proceedings of the National Academy of Sciences of the United States of America*.

[20] Ye Q., Wu Y. L., Foley L. M. (2008). Longitudinal tracking of recipient macrophages in a rat chronic cardiac allograft rejection model with noninvasive magnetic resonance imaging using micrometer-sized paramagnetic iron oxide particles. *Circulation*.

[21] Bernas L. M., Foster P. J., Rutt B. K. (2007). Magnetic resonance imaging of in vitro glioma cell invasion. *Journal of Neurosurgery*.

[22] Raschzok N., Morgul M. H., Pinkernelle J. (2008). Imaging of primary human hepatocytes performed with micron-sized iron oxide particles and clinical magnetic resonance tomography. *Journal of Cellular and Molecular Medicine*.

[23] Williams J. B., Ye Q., Hitchens T. K., Kaufman C. L., Ho C. (2007). MRI detection of macrophages labeled using micrometer-sized iron oxide particles. *Journal of Magnetic Resonance Imaging*.

[24] Vaithilingam V., Barbaro B., Oberholzer J., Tuch B. E. (2011). Functional capacity of human islets after long-distance shipment and encapsulation. *Pancreas*.

[25] Vaithilingam V., Kollarikova G., Qi M. (2011). Effect of prolonged gelling time on the intrinsic properties of barium alginate microcapsules and its biocompatibility. *Journal of Microencapsulation*.

[26] Appavoo M., Tuch B. (2009). Effect of upregulation of NeuroD in insulin-producing liver cells. *Islets*.

[27] Sidhu K., Kim J., Chayosumrit M., Dean S., Sachdev P. (2012). Alginate microcapsule as a 3D platform for propagation and differentiation of human embryonic stem cells (hESC) to different lineages. *Journal of Visualized Experiments*.

[28] Evgenov N. V., Medarova Z., Dai G., Bonner-Weir S., Moore A. (2006). In vivo imaging of islet transplantation. *Nature Medicine*.

[29] Frank J. A., Miller B. R., Arbab A. S. (2003). Clinically applicable labeling of mammalian and stem cells by combining superparamagnetic iron oxides and transfection agents. *Radiology*.

[30] Oca-Cossio J., Mao H., Khokhlova N. (2004). Magnetically labeled insulin-secreting cells. *Biochemical and Biophysical Research Communications*.

[31] Strand B. L., Ryan L., In't Veld P. (2001). Poly-L-lysine induces fibrosis on alginate microcapsules via the induction of cytokines. *Cell Transplantation*.

[32] Tam S. K., De Haan B. J., Faas M. M., Hallé J.-P., Yahia L., De Vos P. (2009). Adsorption of human immunoglobulin to implantable alginate-poly-L-lysine microcapsules: effect of microcapsule composition. *Journal of Biomedical Materials Research—Part A*.

[33] Shapiro E. M., Sharer K., Skrtic S., Koretsky A. P. (2006). In vivo detection of single cells by MRI. *Magnetic Resonance in Medicine*.

[34] Arbab A. S., Bashaw L. A., Miller B. R. (2003). Characterization of biophysical and metabolic properties of cells labeled with superparamagnetic iron oxide nanoparticles and transfection agent for cellular MR imaging. *Radiology*.

[35] Weissleder R., Stark D. D., Engelstad B. L. (1989). Superparamagnetic iron oxide: pharmacokinetics and toxicity. *American Journal of Roentgenology*.

[36] Wyss M., Froehlich J. M., Patak M. A. (2007). Gradient-enhanced volume rendering: an image processing strategy to facilitate whole small bowel imaging with MRI. *European Radiology*.

[37] Martí-Bonmatí L., Graells M., Ronchera-Oms C. L. (1996). Reduction of peristaltic artifacts on magnetic resonance imaging of the abdomen: a comparative evaluation of three drugs. *Abdominal Imaging*.

