# Supplementary material for: Noninvasive Tracking of Encapsulated Insulin Producing Cells Labelled with Magnetic Microspheres by Magnetic Resonance Imaging
Source: J Diabetes Res. 2016 Aug 18;2016:6165893. doi: 10.1155/2016/6165893 (PMC5007365; doi:10.1155/2016/6165893)
Supplement: Supplementary file 1 — The entrapment of MM within alginate microcapsules for varied time points, in vivo MRI of encapsulated MM labelled cells by 3T scanner and movie files for in vivo MRI of encapsulated MM labelled cells by 11.7T scanner are provided in the Supplementary Material [file 6165893.f1.docx]

**
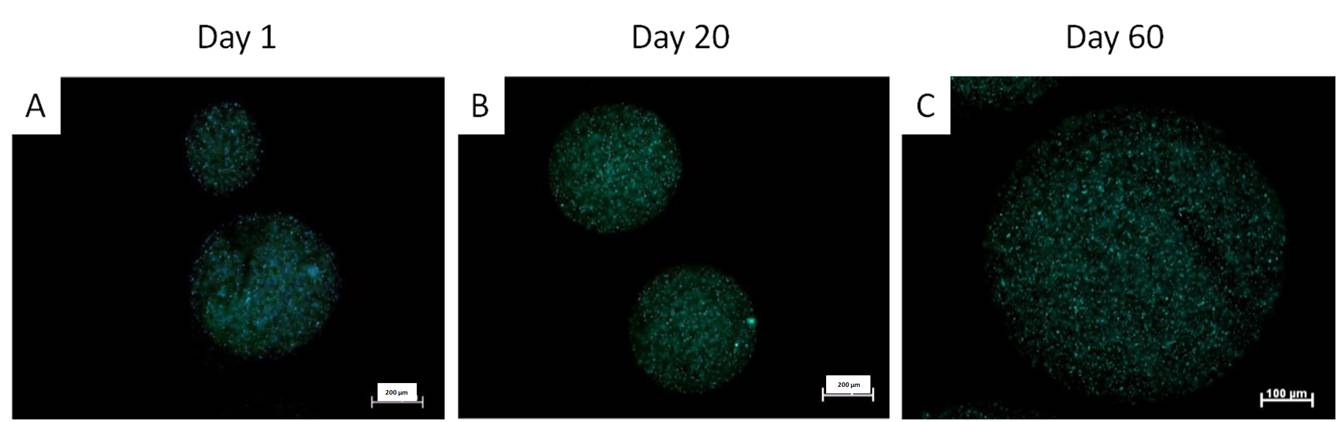
**

**Supplementary Figure 1. Encapsulation of MM in alginate microcapsules.** The MM remained trapped within the alginate microcapsules with no evident leaching when cultured in phosphate buffered saline for days 1, 20 and 60 respectively. Bars are 200 µm for A&B and 100 µm for C.

**
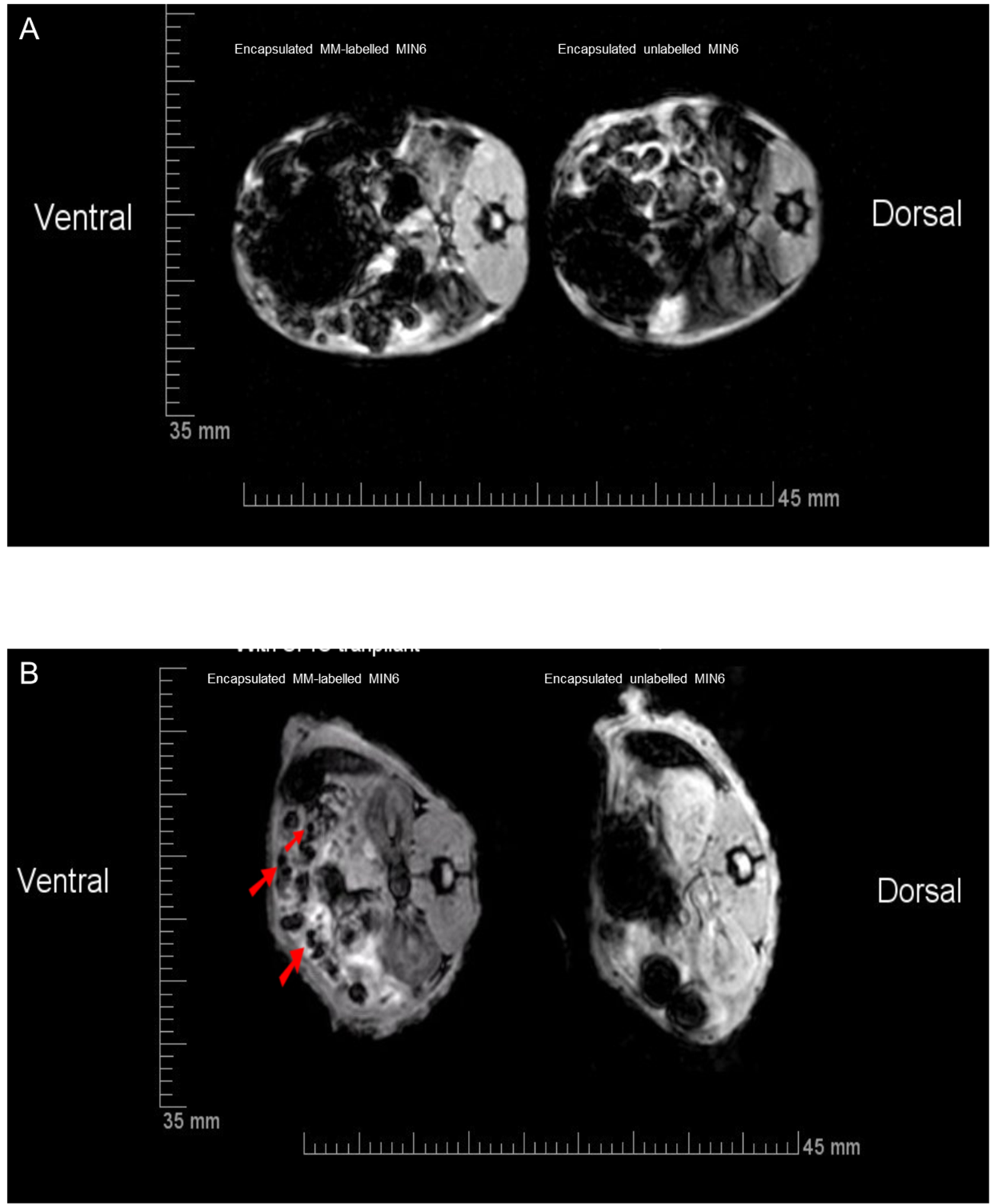
**

**Supplementary Figure 2. *In vivo* MRI of encapsulated MM-labelled cells by 3T scanner.** T2*-weighted images of the abdominal cavity of mice transplanted with encapsulated MM-labelled MIN6 scanned without (A) and with (B) administration of 50% v/v glucose and hypotonic agent Buscopan^®^. Encapsulated MM-labelled MIN6 can only be visualised as dark hypointensities (red arrows) after administration of glucose and hypotonic agent Buscopan^®^.

**Supplementary movie files**
